# Supplementary material for: Novel Intronic Mutation in VMA21 Causing Severe Phenotype of X-Linked Myopathy with Excessive Autophagy—Case Report
Source: Genes (Basel). 2022 Nov 29;13(12):2245. doi: 10.3390/genes13122245 (PMC9777698; doi:10.3390/genes13122245)
Supplement: Supplementary file 1 [file genes-13-02245-s001.zip › genes-2024714-supplementary.pdf]

Supplementary Material and Method :

Cell culture.

Wild type fibroblast control lines AB249 and V972 were derived at CBC biotec (HCL, Lyon) from skin biopsy from a young male control patient of 28 and 23 years old respectively. VMA21 patient fibroblasts AC969 was also derived from skin biopsy at 18 years old. Fibroblasts were cultured in Ham F10 medium supplemented with 12% FBS and antibiotics. Cells were passed once a week

QRT-PCR

Total RNAs were extracted with the RNeasy mini Kit from Qiagen (ref 74104) with DNase on column digestion following provider's instructions and quantified with the nanodrop (ThermoFisher). To generate cDNA, total RNA was reverse transcribed with RevertAid H Minus Reverse Transcriptase (ThermoFisher) primed with random hexamers. RT–qPCR was performed using SYBR Green SuperMix (Biorad, SsoAdvanced Universal SYBR Green Supermix) in the CFX-connect system (BioRad). QPCRs were performed as follows 95°C 3 min to activate the polymerase (SsoAdvanced Universal SYBR Green Supermix) followed by 39 cycles at 95°C for 3 sec and 60°C for20 sec. Relative expression levels were normalized with the mean of *HPRT*, *TBP,* and *GAPDH* housekeeping genes using the $\Delta\Delta C_t$ method. Primers are listed here. HPRT for GACACTGGCAAAACAATGCA ; HPRT rev GGTCCTTTTCACCAGCAAGCT ; GAPDH for GAAGGTGAAGGTCGGAGTC ; GAPDH rev GAAGATGGTGATGGGATTC ; TBP for CACGAACCACGGCACTGATT ; TBP rev TTTCTTGCTGCCAGTCTGGAC ; VMA21-201 Exon 1-2 for GATAAGGCGGCGCTGAAC ; rev AACAGGAGCGTCTTCAGTGT; VMA21-202 Exon 2-3 for TACTCGCCAGGCCTGCTC ; rev AACAGGAGCGTCTTCAGTGT; VMA21 all isoforms for AGACGCTCCTGTTCTTCACA ; rev : ATACACAAAGAGGGCCAGCA.

In order to quantify intron retention, qPCRs were done on the same cDNA reverse transcript from the same total RNA. qPCR was performed as follows 98°C 3 min to activate the polymerase (ONEGreen Fast qPCR Premix, Ozyme) followed by 45 cycles at 95°C for 5 sec and 60°C for30 sec, then followed by the standard melting curve for quality control. Primers used were designed using Primer3 and were validated experimentally by confirming that (i) they amplified a unique band, (ii) they generated a unique peak in their melting curve, and (iii) when the template was diluted, the amplification was proportional to the dilution. The primers that didn't meet these criteria were rejected. The validated primer couple is VMA21 intron forward CTACACTGAAGACGCTCCTG and reverse TAATGGGTCTAAGATTACCTTCA. These primers generate an amplicon of 108 pb. As described above, relative expression levels were normalized with housekeeping genes using the ΔΔCt method.

Western-Blot

Total cell extracts were obtained by directly solubilizing the cells in 400µl 1X Laemmli buffer (50mM Tris HCl pH 6.8, 10% glycerol, 100mM DTT, 2% SDS, Bromophenol blue) supplemented with 30U benzonase (ThermoFisher ref 70664) per 2.10^6 cells. After 15 min incubation at room temperature, extracts are scrapped from the culture plate, placed in a 1.5ml tube, boiled for 7 min, and directly loaded on an AnykD gel from Biorad. Once the complete migration is obtained gels are transferred onto a Nitrocellulose membrane (0.22µm, Amersham)) and blotted with the indicated antibodies. VMA21 Polyclonal Antibody Thermo Fisher PA5-42630 1/500. Anti-Histone H4 Antibody, pan, rabbit monoclonal Merck millipore 04-858, 1/5000. Anti-α-Tubulin antibody, Mouse monoclonal Sigma Aldrich T6074, 1/5000

Supplementary figure

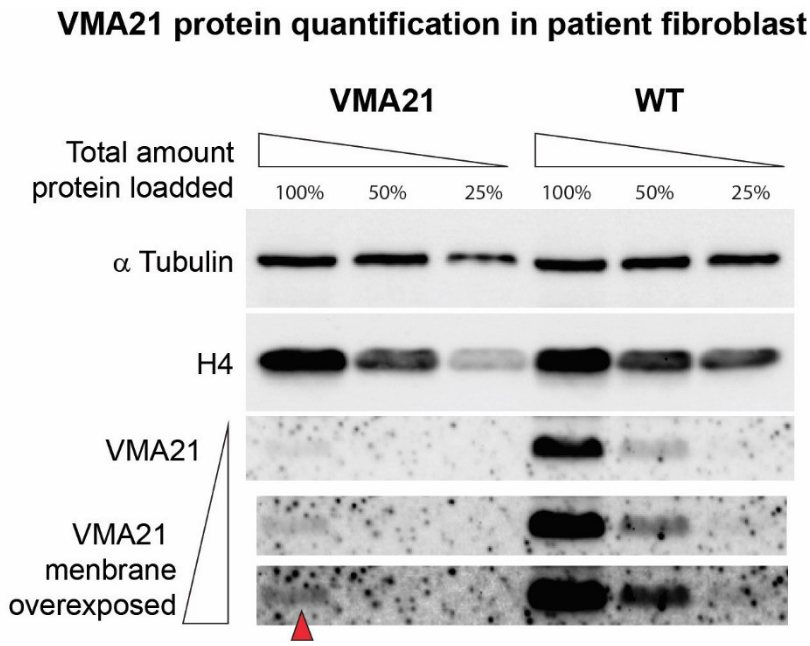

Supplementary Figure S1 VMA21 western blot quantification in patient fibroblast

Membrane overexposed for VMA21 showing weak signal (red arrow) in patient fibroblast that could be compare to the control VMA21 fibroblast and correspond to a signal between 50% to 25% of the control.
